# Structural Insights into the Methylation of C1402 in 16S rRNA by Methyltransferase RsmI

**DOI:** 10.1371/journal.pone.0163816

**Published:** 2016-10-06

**Authors:** Mohan Zhao, Heng Zhang, Guangfeng Liu, Li Wang, Jian Wang, Zengqiang Gao, Yuhui Dong, Linbo Zhang, Yong Gong

**Affiliations:** 1 Beijing Synchrotron Radiation Facility, Institute of High Energy Physics, Chinese Academy of Sciences, Beijing, China; 2 College of Life Science, Jilin Agricultural University, Changchun, China; 3 National Center for Protein Science Shanghai, Institute of Biochemistry and Cell Biology, Shanghai Institutes for Biological Sciences, Chinese Academy of Sciences, Shanghai, China; University of Washington, UNITED STATES

## Abstract

RsmI and RsmH are conserved S-Adenosylmethionine (AdoMet)-dependent methyltransferases (MTases) that are responsible for the 2′-*O*-methylation and N^4^-methylation of C1402 in bacterial 16S rRNA, respectively. Methylation of m^4^Cm1402 plays a role in fine-tuning the shape and functions of the P-site to increase the decoding fidelity, and was recently found to contribute to the virulence of *Staphylococcus aureus* in host animals. Here we report the 2.20-Å crystal structure of homodimeric RsmI from *Escherichia coli* in complex with the cofactor AdoMet. RsmI consists of an N-terminal putative RNA-binding domain (NTD) and a C-terminal catalytic domain (CTD) with a Rossmann-like fold, and belongs to the class III MTase family. AdoMet is specifically bound into a negatively charged deep pocket formed by both domains by making extensive contacts. Structure-based mutagenesis and isothermal titration calorimetry (ITC) assays revealed Asp100 and Ala124 are vital for AdoMet-binding. Although the overall fold of RsmI shows remarkable similarities to the characterized MTases involved in vitamin B12 biosynthesis, it exhibits a distinct charge distribution especially around the AdoMet-binding pocket because of different substrate specificity. The docking model of RsmI-AdoMet-RNA ternary complex suggested a possible base-flipping mechanism of the substrate RNA that has been observed in several known RNA MTases. Our structural and biochemical studies provide novel insights into the catalytic mechanism of C1402 methylation in 16S rRNA.

## Introduction

Methylation of ribosomal RNA (rRNA) by methyltransferases (MTases) is closely associated with the fine-toned protein synthesis in ribosome and related physiological processes, such as 30S subunit assembly, fine-tuning of local rRNA structure and antibiotic resistance in some cases [1–6]. 2′-*O*-methylation and pseudouridylations usually occur in eucaryote and archaea, whereas base methylation is most common in bacteria [7, 8]. The methylated nucleosides in rRNA are mostly clustered within and around the conserved decoding and peptidyltransferase active sites and can modulate translational fidelity of ribosome [7]. RsmI and RsmH were recently identified S-Adenosylmethionine (AdoMet)-dependent MTases responsible for the 2′-*O*-methylation and N^4^-methylation of C1402 (m^4^Cm1402) in bacterial 16S rRNA, respectively [9] (S1 Fig). The position C1402 can make direct contacts with the phosphate backbone of the P-site codon, and the modification by RsmI and RsmH plays an important role in P-site function, especially in start codon selection. The P-site contains several conserved nucleotides (positions 1400–1405 and 1496–1502) in the top part of helix 44 in the 16S rRNA of the 30S subunit [10].

The *rsmI* and *rsmH* genes are conserved in nearly all species of bacteria, and their homologs are also discovered in several species of eukaryotes, suggesting that this modification at the P-site is a common structural feature of bacterial 16S rRNA [9]. Both single and double knock-out strains of Δ*rsmI/*Δ*rsmH* can cause growth reduction compared with the wild-type in *Escherichia coli* [9], as observed in the effect of several point mutations in the P-site previously [11, 12]. The lack of either methylation of C1402 was found to affect translational initiation efficiency and decrease the UGA read-through rate [9]. Therefore, m^4^Cm1402 may play a role in fine-tuning the shape and function of the P-site to increase the decoding fidelity. The 30S subunit, rather than protein-free 16S rRNA, can function as the substrate of recombinant RsmI and RsmH, suggesting that this modification is formed at a late step during 30S assembly *in vivo* [9]. Moreover, a recent study showed m^4^Cm1402 contributes to the virulence of *Staphylococcus aureus* by conferring resistance to oxidative stress in host animals [13].

Recently, we reported the crystal structure of RsmH in complex with AdoMet and cytidine, which showed that the structural rearrangement of RsmH or the nucleotides around C1402 may be necessary to trigger catalysis in the methylation [14]. However, the three-dimensional structure of RsmI remained unknown. RsmI is the only 2′-*O*-MTase among known MTases modifying bacterial 16S rRNA, and its catalytic mechanism remains unclear. Here we reported the high resolution crystal structure of *E*. *coli* RmsI in a dimer form in complex with AdoMet. By structure comparisons, cofactor binding analyses, structure-based mutagenesis and ITC assays, we revealed the structure-function relationship of RsmI. Furthermore, a docking model of RsmI-AdoMet-RNA ternary complex is proposed to guide future investigations on the process of C1402 dimethylation in 16S rRNA.

## Results

### Overall structure

Our previous study showed the full-length RsmI was not amenable to crystallization [15], and the truncation Gly12-Pro258 was expressed, purified and co-crystallized with the AdoMet substrate in this study. The complex structure was solved by molecular replacement in space group C2 at a 2.20 Å resolution, and the details of data collection and structure refinement are given in Table 1. The asymmetric unit contains three RsmI molecules; two of them form a dimer formed by a noncrystallographic two-fold axis, while the third one also participates in a dimer, formed by the crystallographic two-fold axis (S2 Fig).

**Table 1:** Data collection and refinement statistics.

	RmsI-AdoMet complex
**Data collection**	
Space group	C2
Cell dimensions	
*a*, *b*, *c* (Å)	120.9, 155.3, 54.6
α, β, γ (°)	90.0, 93.9, 90.0
Wavelength (Å)	0.98
Resolution (Å)	50–2.2 (2.24–2.20) ^a^
*R*_merge_^b^	0.063 (0.535)
〈*I*/σ(*I*)〉	31.5 (4.1)
Completeness (%)	93.1(97.7)
Redundancy	3.2
**Refinement**	
Resolution (Å)	50–2.2
No. reflections	46,145
*R*_work/_ *R*_free_	0.203/0.230
No. atoms	
Protein	5,190
Ligands	81
Water	311
B-factors	53.0
R.m.s deviations	
Bond lengths (Å)	0.008
Bond angles (°)	1.1
Ramachandran statistics^c^, residues in (%)	
Most favoured regions	98.6
Allowed regions	1.4
Disallowed regions	0

^a^ The values in parenthesis mean those of the highest resolution shell.

^b^ Correlation coefficient for data in the highest resolution shell only.

^c^ Ramachandran statistics were calculated using MolProbity.

**Fig 1:**
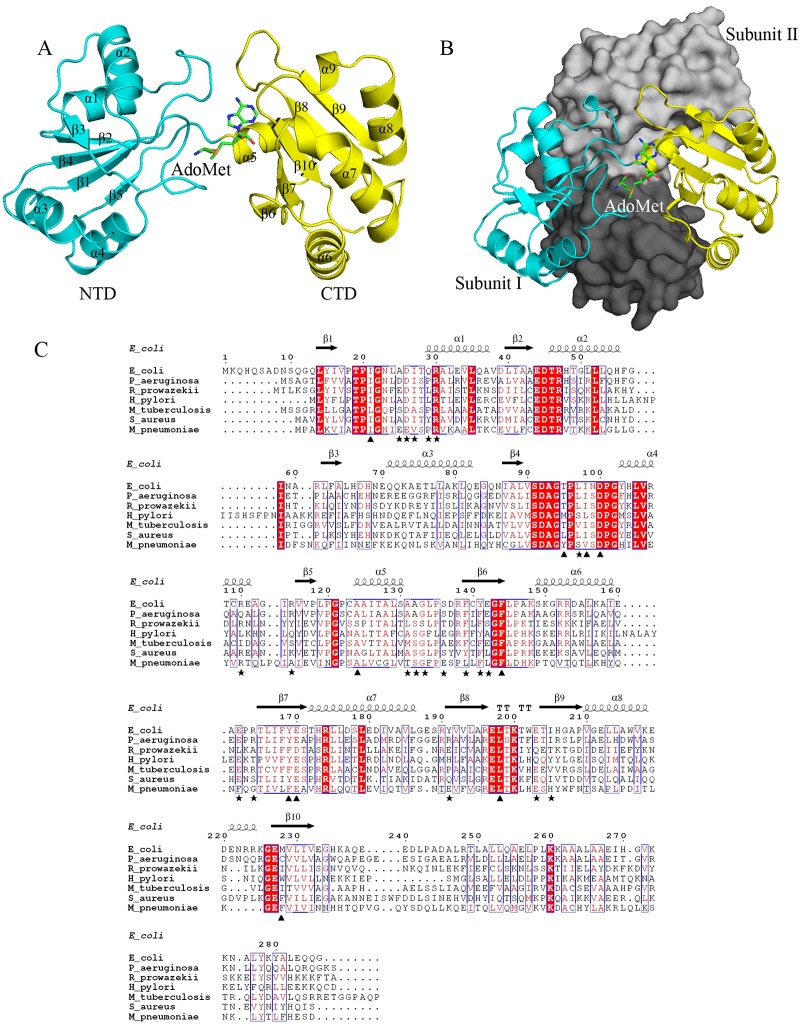
Overall structure of RsmI-AdoMet complex. (A) Cartoon representation showing the domain architecture of RsmI with NTD in cyan and CTD in yellow. The binding AdoMet molecule is shown in green sticks. (B) Dimeric structure of RsmI, with subunit I and II shown in cartoon and surface, respectively. The NTD and CTD of subunit II are shown in light and dark gray, respectively. The two subunits make contacts by a “back to back” mode and the AdoMet-binding site is exposed. (C) Structure-based sequence alignment for representative RsmI family members from gram negative and positive bacteria, including *E*. *coli*, *Pseudomonas aeruginosa* (P_ aeruginosa), *Rickettsia prowazekii* (R_prowazekii), *Helicobacter pylori* (H_pylori), *Mycobacterium tuberculosis* (M_tuberculosis), *Staphylococcus aureus* (S_aureus) and *Mycoplasma pneumoniae* (M_pneumoniae), performed using clustal X (version 1.81) and ESPript 3.0 [31]. The conserved residues are boxed in blue. Identical conserved and low conserved residues are highlighted in red background and red letters, respectively. The residues involved in RsmI dimer formation are labelled using stars and those involved in AdoMet-binding are labelled using triangles.

The structure of RsmI monomer is composed of two independent domains: the N-terminal domain (NTD) and the C-terminal domain (CTD) (Fig 1A and 1C). The two domains are connected by a short linker region, and there is no topological similarity between them. NTD is a putative RNA-binding domain (discussed below), formed by the residues from Gly12 to Gly121. This domain consists of five parallel β-strands and four helices forming a mixed α/β fold, described as β1, α1, β2, α2, β3, α3, β4, α4 and β5, with the strands in the center and the helices on the sides. CTD is a conserved MTase catalytic domain with a Rossmann-like fold, formed by the residues from Pro122 to Glu240. The MTase domain consists of five twisted anti-parallel β-strands flanked by five helices described as α5, β6, α6, β7, α7, β8, β9, α8, α9 and β10.

### Dimerization of RsmI

The gel filtration chromatography showed that RsmI is likely to be dimeric in solution (S3 Fig). The crystal structure of RsmI further reveals a compact homodimer by making extensive contacts between two subunits (Fig 1B). The two subunits interact with each other by a “back to back” mode and are nearly perpendicular with each other, and in this case the AdoMet is exposed to solvent for catalysis. The buried surface area in the non-crystallographic dimer interface is 2,102 Å^2^, which is similar to that (2,094 Å^2^) in the crystallographic dimer. The direct interactions [including hydrogen bonds (H-bonds) and salt bridges within 3.9 Å) in the dimer interface are also largely similar in the two types of dimer (S1 Table). The interacting residues are mainly located in α4-α6, β5-β8 and their connecting loops, most of which are highly conserved in RsmI homologs (Fig 1C). This suggests that molecular dimerization may be required for the catalysis of RsmI, as observed in its homologs precorrin MTases [16, 17] (discussed below), and most of 16S rRNA MTases [14, 18].

### Structural comparisons of RsmI with related proteins

**Fig 2:**
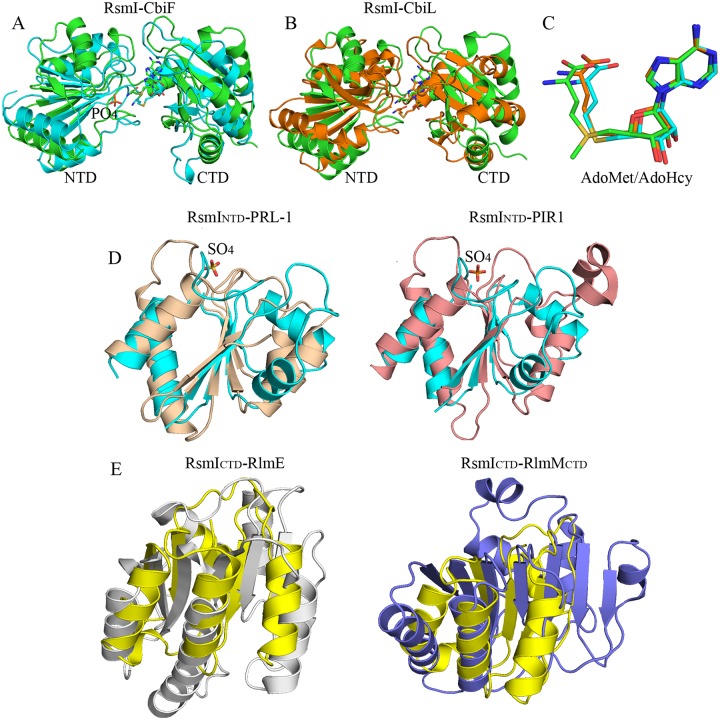
Structure comparisons of RsmI with its homologs. (A-B) Structural superimpositions of RsmI (green) with representative members of AdoMet-dependent MTases involved in cobalamin biosynthesis, including CbiF (cyan, PDB code 1CBF) and CbiL (orange, PDB code 2E0N). One phosphate molecule (orange sticks) is bound to the proposed substrate binding site in NRD that is close to the AdoHcy molecule in CbiF. (C) Structural superimpositions of AdoMet (green) with AdoHcy in CbiF (cyan) and CbiL (orange), showing similar bent conformations in class III MTases family. (D) Structural superimpositions of RsmI NTD (cyan) with representative members of protein tyrosine phosphatase (PTP) superfamily, PRL-1 (wheat, PDB code 1XM2) and PIR1 (pink, PDB code 4NYH). The active-site bound sulfate ion (orange sticks) in PTPs is known to mimic the substrate phosphate group. (E) Structural superimpositions of RsmI CTD (yellow) with rRNA MTases RlmE (gray, PDB code 1EIZ) and RlmM (purple, PDB code 4B17). RsmI CTD adopts a Rossmann-like fold distinguished from those of RlmE and RlmM that belong to class I MTase family.

According to the DALI search (http://ekhidna.biocenter.helsinki.fi/dali_server), the overall structure of RsmI shows significant structural similarity with several predicated MTases solved by structural genomics consortia (with Z-scores of ~20, data not shown), thus far without published functional analyses. RsmI has a notably similar overall fold with several characterized AdoMet-dependent MTases involved in anaerobic vitamin B12 biosynthesis, such as cobalt-precorrin-4-MTase CbiF from *Bacillus megaterium* (PDB code 1CBF, with Z-score 17.3 and r.m.s.d. 3.5 Å for 209 Cα atoms), cobalt-precorrin-2 C20-MTase CbiL from *Chlorobium tepidum* (PDB code 2E0N, with Z-score 16.7 and r.m.s.d. 3.6 Å for 203 Cα atoms) (Fig 2A–2C). They belong to the class III MTase family despite their low sequence identities (18% and 17% identity, respectively) [19]. One phosphate molecule is bound to the proposed substrate binding site in NRD of CbiF, which is close to the S-Adenosylhomocysteine (AdoHcy) molecule (Fig 2A), suggesting the NTD in RsmI is also involved in substrate recognition. Moreover, the bent conformation of AdoMet in RsmI is also remarkably similar to AdoHcy in CbiF and CbiL (Fig 2C, with less than 0.9 Å r.m.s.d.), which is a specific conformation among the class III MTase family members with a common catalytic domain structure [19]. Although the overall fold of RsmI shows remarkable similarity to the characterized MTases involved in vitamin B12 biosynthesis, they have different distinct surface charge distribution especially in the region around AdoMet-binding pocket (S4 Fig). The distinct positive charge distributions reflect their diverse functions in recognizing specific substrates.

The DALI search and structural comparison also showed RsmI NTD has a significantly similar fold to several members of protein tyrosine phosphatase (PTP) superfamily, such as PRL-1 (phosphatase of regenerating liver) (PDB code 1XM2, with r.m.s.d. 3.4 Å for 88 Cα atoms) and PIR1 (phosphatase that interacts with RNA-ribonucleoprotein complex 1) (PDB code 4NYH, with r.m.s.d. 3.2 Å for 79 Cα atoms) (Fig 2D). Moreover, the active-site bound sulfate ion in PTPs is known to mimic the substrate phosphate group [20], suggesting the similar role of NTD of RsmI in RNA-binding. Structural comparisons of RsmI CTD with the MTases RlmE (PDB code 1EIZ) and RlmM (PDB code 4B17) that belong to class I MTase family showed RsmI adopts a distinct Rossmann-like fold (Fig 2E), although all of them are responsible for 2’-*O*-ribose methylation. The canonical class I Rossmann-like MTase fold consists of a mixed seven-stranded β-strands flanked by six helices [19], whereas there are five-stranded β-strands (β6-β10) flanked mixed by five helices (α5-α9) in RsmI CTD (Fig 1A).

### AdoMet binding in the active site

**Fig 3:**
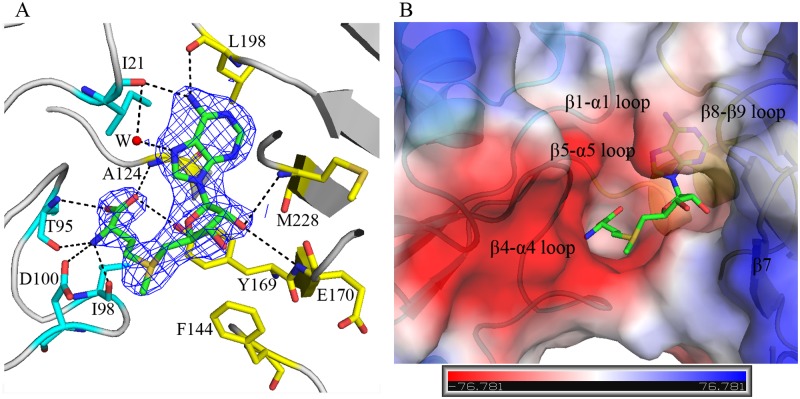
AdoMet binding in the active site formed by NTD and CTD. (A) Contacts analysis between AdoMet (green sticks) and RsmI (gray cartoon). The interacting residues from NTD and CTD are highlighted using cyan and yellow sticks, respectively. A water molecule (W) is shown as red sphere. Electron density map (2*F*_*o*_*-F*_*c*_) of AdoMet is contoured at a 2σ level. (B) AdoMet binds into a deep pocket formed by several flexible loops from both NTD and CTD (shown as surface electrostatic potential, blue, +7.8KT; red, -7.8KT), with dominantly negative charges.

AdoMet is bound into the cleft between NTD and CTD in the present complex, and the electron density for the whole AdoMet molecule was clear (Fig 3A), therefore with well-defined conformation and orientation. AdoMet is bound tightly in a canonical conformation in the pocket which mainly consists of three loops: β1-α1 and β4-α4 from NTD domain, and β8-β9 from CTD domain, as well as sheet β7, forming a deep groove with dominantly negative charges (Fig 3B). The cavity is large enough to accommodate a cytidine ring and position its methylated 2’-hydroxyl group next to the active site residues. The main part of the methionine side chain of AdoMet inserts into the large cavity, whereas the reactive methyl group of sulfur atom is oriented outside for substrate-binding, while the adenosine ring overhangs into the large cavity (Fig 3B).

The direct contacts (within 3.8 Å) with AdoMet are mediated by Ile21, Thr95, Ile98 and Asp100 from NTD, and Ala124, Tyr169, Glu170, Leu198 and Met228 from CTD, with twelve H-bonds that stabilize the position and orientation of AdoMet (Fig 3A). Most of these residues are conserved in RsmI orthologs (Fig 1C). The direct contacts with AdoMet are mediated by the main chains of these residues in RsmI, except the side chains of Asp100 (OD2, 2.9 Å) and Y169 (hydroxyl, 3.4 Å) interacting with AdoMet. The AdoMet sulfur atom is approached by D100 and F144. The methionine of AdoMet is stabilized by its amino group interacting with Thr95, Ile98 and Asp100 by forming three H-bonds, and by its carboxyl group with Ala124 and Tyr169, as well as its hydroxyl group with Thr95. The N6 atom of the adenine group is coordinated by the H-bonds to Ile21 (2.9 Å) and Leu198 (2.9 Å), while the N7 atom contacts with the main chain of Ile21 indirectly via a well-ordered water molecule.

### Binding characterics of RsmI to AdoMet and the putative substrate

**Fig 4:**
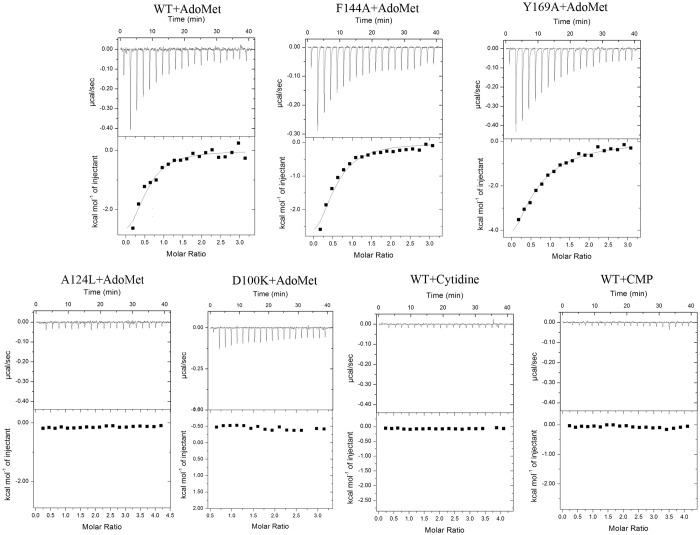
ITC spectra for the binding of RsmI wild-type and mutants to AdoMet/cytidine/CMP. Baseline subtracted raw ITC data for injections of AdoMet (cytidine or CMP) is indicated in the upper panels of each of the ITC profiles shown (for the wild-type as well as the variants of RsmI). The peaks normalized to the ligand/protein molar ratio were integrated as is shown in the bottom panels. The solid dots indicate the experimental data and the best fit to the experimental data were obtained from a non-linear least squares method of fitting using a one-site binding model depicted by a solid line. The binding affinity of each mutant is from the average of three independent experiments.

The interaction of wild-type RsmI with AdoMet was characterized by ITC assay (Fig 4 and Table 2). The integrated heat data could be fitted well using the one-site model and RsmI concentration was quantified in the monomeric form, with a binding affinity (Ka) of 4.29 ×10^4^ M^-1^ in a heat-releasing process (ΔH = -5.56 kcal/mol). In contrast, our previous study on 16S rRNA MTase RsmE showed the first AdoMet-binding affinity is significantly higher than the second one (9.44 ×10^4^ M^-1^ *vs* 0.074 ×10^4^ M^-1^), indicating the binding may be competitive causing only one AdoMet binding by active dimeric RsmE [18]. The AdoMet-binding characterics of RsmE can only be fitted using a sequential binding sites model, but cannot using one-site or two-site model, and in this case its concentration was quantified in the dimeric form.

**Table 2:** ITC data for titration of RsmI variants with AdoMet and putative substrates fitting using a one-site binding model.

RsmE binding	*Ka* (10^4^/M)	*ΔH* (Kcal/mol)	*-TΔS* (Kcal/mol)	*ΔG* (Kcal/mol)
WT/AdoMet	4.29 ± 0.84	-5.56 ± 0.42	-0.051	-5.61
F144A/AdoMet	1.48 ± 0.16	-22.09 ± 1.61	1.10	-20.99
Y169A/AdoMet	4.14 ± 0.60	-6.50 ± 0.75	0.014	-6.49
D100K/AdoMet	NB			
A124L/AdoMet	NB			
WT/Cytidine	NB			
WT/CMP	NB			

Ka: binding affinity; ΔH: change in enthalpy; -TΔS: change in entropy; ΔG: Gibb's free energy.

NB: no detectable binding.

Further structure-based mutagenesis of RsmI was performed to confirm the key residues involved in AdoMet-binding. According to our crystal structure, we predicted the mutant D100K would abolish its side chain-mediating H-bond with AdoMet and significantly alter the surface change of AdoMet-binding pocket (from negative to positive). It was confirmed by the ITC result of D100K mutation with a complete loss of binding affinity (Fig 4 and Table 2). So it reveals that Asp100 is a vital residue for the substrate binding and methyltransferase activity of RsmI. Moreover, the ITC results showed that the mutant A124L also completely abolished the H-bond with AdoMet. It should be caused by the obvious steric hindrance of the sidechain of leucine side with the AdoMet adenosine ring in the binding pocket, as shown in the A124L mutant model of RsmI (S5 Fig). In addition, the binding affinities and entropy of F144A were moderately affected compared with those of the wild-type, indicating that the hydrophobic interaction medicated by Phe144 also plays a relatively important role in AdoMet-binding. Meanwhile, the binding characters of Y169A, including the binding affinity and enthalpy/entropy, were very similar to that of the wild-type, indicating neither the H-bond nor the hydrophobic interaction by Tyr169 is necessary for AdoMet-binding.

Unexpectedly, RsmI shows no detectable binding to the mimic substrates cytidine or CMP in the ITC experiments (Fig 4), suggesting that it requires the fully assembly 30S subunit as the real substrate [9]. It also explains why we did not find the density of CMP in the crystal structure of RsmI when cocrystallized with AdoMet and CMP.

### Docking model of RsmI-AdoMet-rRNA complex

**Fig 5:**
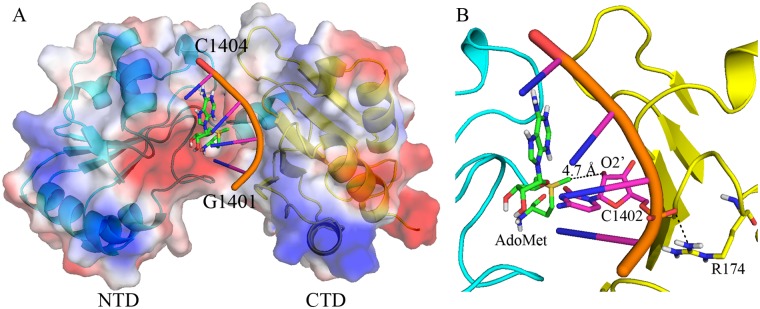
Docking model of RsmI-AdoMet-rRNA ternary complex. (A) A molecular surface and cartoon representation of RsmI, colored by its local electrostatic potential. The substrate RNA is bound into the cleft between NTD (in cyan) and CTD (in yellow) and close to AdoMet (green sticks). AdoMet and C1402 are shown in green and magenta sticks, respectively. (B) A detailed view of the interactions in the model. Arg174 (yellow sticks) can form an H-bond with the phosphate backbone linking C1402. The distance between the AdoMet methyl group and the target O2’ atom of C1402 is 4.7 Å, which may require a slight shift to trigger catalysis.

Since the role of the ribosomal proteins (r-proteins) in the substrate recognition by RsmI remains unknown, we have docked a model of RsmI-AdoMet-rRNA ternary complex, using the rRNA fragment (G1401-C1404) from the structure of *E*. *coli* 30S subunit (PDB code 2AW7) as the putative substrate. The molecular dynamics analysis indicated that the model was both structurally feasible and energetically stable. The minimized model showed that the RNA loop likely fit into the cleft generated by the NTD and CTD easily, and bind to the region mainly distributed with positive charge (Fig 5A). In 30S subunit structure, the RNA fragment C1400-G1405 forms base pairs with neighboring nucleotides C1496-C1501 in helix 44). Therefore, C1402 possibly firstly unfolded from the Watson-Crick pairing with neighboring A1500 to a single-stranded conformation. Then C1402 may flip out of the helix 44 to insert into the active site of RsmI, accompanied by the significant conformational changes.

In this model, the distance between the AdoMet methyl group and the substrate O2’ atom is 4.7 Å (Fig 5B), indicating the cytidine (C1402) is bound in a catalytically inactive state, and a small shift may be sufficient to trigger the catalysis. Moreover, the side chain of the highly conserved residues Arg174 can form an H-bond with the phosphate backbone linking C1402 to stabilize the conformation (Fig 5B). Meanwhile, there is a large positively charged surface area surrounding the AdoMet-binding pocket (Fig 5A), which may mediate the binding of the negatively charged substrate RNA near the active site of RsmI.

## Discussion

There are several RNA MTases with modifications clustered around the decoding center, such as RsmC, RsmE and RsmH/ RsmI, which universally require the assembled 30S as substrate in *E*. *coli* [6]. Our docking model suggests a possible base-flipping mechanism of the target in rRNA structure for RsmI. The induced-fit mechanism has also been observed in the 16S rRNA MTase RsmC, with the target G1207 disengaging from C1051 and flipping out into the active site prior to its modification [21]. The pseudouridine (Ψ) synthase TruB-RNA structure showed this enzyme recognizes the preformed three-dimensional structure of the T loop, and it accesses its substrate uridyl residue by flipping out the nucleotide and disruption of tRNA tertiary structure [22]. A recent report showed the crystal structure of novel plasmid-mediated aminoglycoside-resistance rRNA MTase A (NpmA) in complex with its substrate 30S subunit in a “precatalytic state”, which modifies A1408 in helix 44 of 16S rRNA adjacent to the decoding center [23]. NpmA binds at the 30S decoding center and interacts with four 16S rRNA helices (helix 24, 27, 44 and 45). A1408 is detached from the H-bond to A1493, and flipped out with a rotation ∼180° around its helical axis of helix 44. Arg205 and Arg207 in NpmA are likely to promote or stabilize the flipped conformation by making electrostatic interactions at the A1408 phosphate. In our study, C1402 modified by RsmI/RsmH is very close to A1408, both of which are located in the decoding center and require the mature 30S subunit as the substrate. Therefore, considering the remarkable similarities between the two 16S rRNA MTases, we reasonably speculate that RsmI/RsmH will adopt the base-flipping mechanism like NpmA, and may also exploit features of 16S rRNA helices (such as helix 44/45) tertiary surface to achieve the target recognition and specificity. Meanwhile, the real substrate of RsmI, consisting of rRNA and r-proteins *in vivo*, is highly structured and more complicated, and its methylation mechanism may be somewhat different from NpmA. Moreover, C1402 is located at the deep of the groove rather than A1408 in the surface of 30S subunit. This indicates that, unlike the minimal disruption of rRNA structure by NpmA, the structural rearrangement of the access to C1402 may be required and the assembled 30S subunit may undergo significant conformation changes to trigger the catalytic activity of RsmI as well as RsmH.

Interestingly, although both RsmI and RsmH are responsible for the modification of C1402 in bacterial 16S rRNA, they have distinct methylation types (2′-O-methylation and N^4^-methylation of C1402, respectively). Their overall structures are remarkably different with low homology (S6 Fig, the RMSD is up to 4.58), although both of them are composed of two domains. In RsmH-AdoMet-cytidine structure (PDB code 3TKA), the putative substrate cytidine is far (up to 25.9 Å) from the AdoMet in the catalytic pocket. So the cytidine is not in the active status in RsmH structure [14]. In our following studies, we found neither RsmI (Fig 4) nor RsmH (unpublished data) shows detectable binding to the cytidine by ITC assays, as revealed that they require the fully assembly 30S subunit as the real substrate [9].

## Conclusion

In the present study, we reported and compared the crystal structure of RsmI-AdoMet complex. Key residues were also identified by biochemical methods. A deep AdoMet-binding pocket is formed between the putative substrate-binding domain and catalytic domain, and both domains may collaborate in the methylation process of C1402. These results and a proposed docking model of RsmI-AdoMet-rRNA complex may help to further understand the catalytic mechanism of RsmI.

## Materials and Methods

### Cloning and protein expression

The full-length protein and the truncated protein (G12-P258) of *E*. *coli* RsmI were expressed and purified as reported previously [15]. Site-directed mutagenesis of *rsmI* was performed by a PCR-based technique according to the QuikChange site-directed mutagenesis strategy (Stratagene) following the manufacturer’s instructions. The mutant genes were sequenced and found to contain only the desired mutations.

### Protein crystallization

The truncation mutant RmsI (G12-P258) was concentrated to 0.42 mM (13 mg/ml) in a solution containing 50 mM Tris-HCl pH8.5 and 80 mM NaCl. AdoMet (Sigma, USA, 180 mM stock solution) and RmsI were mixed at a molar ratio of 5:1 and incubated on ice for 6 h before performing co-crystallization experiments. The crystallization screen was performed by mixing 1μl RsmI-AdoMet mixture and 1μl well buffer in the 48-well XtalQuest crystallization plate (MiTeGen, USA). Crystals were grown at 291K using the sitting-drop vapor diffusion method. The final crystallization condition is 0.2 M DL-Malic acid (pH 7.0), 20% PEG3350 (Sigma, USA).

### Data collection, crystal structure determination and refinement

Diffraction data were collected on the BL17U beamline of the Shanghai Synchrotron Radiation Facility (SSRF). Before data collection, crystals were soaked for 5 s in a cryoprotectant consisting of 20% (v/v) glycerol in the crystal mother liquid and then vitrified in liquid nitrogen. Data were processed with the program HKL2000 [24].

The initial phases were calculated using the program PHASER [25] with the crystal structure of putative methyltransferase from Lactobacillus brevis (PDB ID: 3KWP) as the searching model. Their sequence identity is 44% and sequence positive is 57%. The translational Z-score values were 13.7, 21.1 and 24.0 for three molecules of the asymmetric unit. The structure refinement was carried out with Refmac and Phenix [26, 27]. Model building was carried out using Coot [28]. MolProbity was used to validate the structure [29]. A summary of data collection and final refinement statistics are listed in Table 1. The program PyMOL (http://www.pymol.sourceforge.net/) was used to prepare structural figures.

### Isothermal titration calorimetry (ITC)

ITC was applied to quantitatively determine the binding affinities of full-length RsmI to AdoMet and the putative substrates, performed as reported previously [18]. For the titration experiments, the protein was purified with the same method as above and dialyzed against the buffer containing 50 mM HEPES (pH 7.5, with Na^+^ concentration 6.9 mM), 0.15 M NaCl and 2 mM DTT for 24 h. The ITC experiments were carried out using a high-sensitivity ITC-200 microcalorimeter from Microcal (GE Healthcare) at 20°C, by titrating a solution of 300–750 μM AdoMet in 20–50 μM RsmI in the sample cell. All samples were thoroughly degassed and then centrifuged to get rid of precipitates. Injection volumes of 2 μl per injection were used, and for every experiment the heat of dilution for each ligand was measured and subtracted from the calorimetric titration experimental runs for the protein. Consecutive injections were separated by 2 min to allow the peak to return to the baseline. Integrated heat data obtained for the ITCs were fitted in a one-site model using a nonlinear least-squares minimization algorithm to a theoretical titration curve, using the MicroCal-Origin 7.0 software package.

### Molecular modeling of RsmI-AdoMet-RNA complex

The coordinates of G1401-C1404 in 16S rRNA were taken from the structure of wild-type *E*. *coli* 30S subunit (PDB code 2AW7) and the structure of RsmI-AdoMet was used as the receptor molecule and calculated with AutoDock 4.2 [30]. Considering some conformation changes of RsmI may take place after bound to the substrate RNA, the AdoMet was treated as a flexible molecule while the ligand (the RNA backbone) was oriented toward the cleft formed by NTD and CTD of RsmI. The grid size is 54×54×54 Å and the grid step is 0.375 Å. Subsequently, both shape-only and shape-electrostatics correlation algorithms were used with a search radius of n = 30, and the top 10 docking solutions were inspected visually in Coot [28]. Solutions from each round of docking were subsequently ranked, according to the proximity between the residues implicated in RsmI binding to the C1402 nucleoside and affinity scores that describe clashes of the ligand with the receptor molecule, and best-scoring poses were regarded as the most likely models. The best packed model (Estimated free energy of binding = -2.37 kcal/mol) was obtained, without any large conformational changes of the protein.

### Protein Data Bank accession code

The atomic coordinate and structure factor of RsmI-AdoMet complex have been deposited with the RCSB PDB with the accession code 5HW4.

## Supporting Information

S1 Fig Modification of C1402 in bacterial 16S rRNA by RsmI and RsmH for the 2′-*O*-methylation and N^4^-methylation, respectively. (TIF)

S2 Fig Crystal packing of the three RsmI molecules (the subunits I, II and III in magenta, cyan and green, respectively) in one asymmetric unit. Three RsmI molecules (the subunits I’, II’ and III’) in another asymmetric unit are shown in gray. The subunits I and II (or I’ and II’) can form a compact homodimer. The subunit III forms another dimer with its crystallographic symmetry molecule III’.
(TIF)

S3 Fig Purified RsmI (Gly12-Pro258) (A) eluted from gel filtration chromatogram (Superdex^™^ 200 10/300 GL) at 15.0 ml, and the elution volume of protein standards (Thermo Scientific, USA) (B) from this column. (TIF)

S4 Fig Comparison of the surface charge distribution of RsmI (+7.8 KT; -7.8 KT), CbiF (PDB code 1CBF, +7.2KT; -7.2KT) and CbiL (PDB code 2E0N, +7.3KT; -7.3KT), by their local electrostatic potential (positive charge in blue and negative charge in red). (TIF)

S5 Fig Surface presentations of wild-type RsmI (A) and the A124L mutant model (B). The adenosine ring of AdoMet shows obvious steric hindrance with L124 in the mutant model, causing its unfavorable binding to AdoMet.
(TIF)

S6 Fig Structural superimposition of RsmI (cyan) with RsmH (magenta, PDB code 3TKA) in ribbon. (TIF)

S1 Table The direct interactions (within 3.9 Å) between the two subunits of RsmI dimer analyzed by PDBe-PISA (http://www.ebi.ac.uk/msd-srv/prot_int/pistart.html). (DOCX)
