# Supplementary figures and images for: Structural Insights into the Methylation of C1402 in 16S rRNA by Methyltransferase RsmI
Source: PLoS One. 2016 Oct 6;11(10):e0163816. doi: 10.1371/journal.pone.0163816 (PMC5053481; doi:10.1371/journal.pone.0163816)

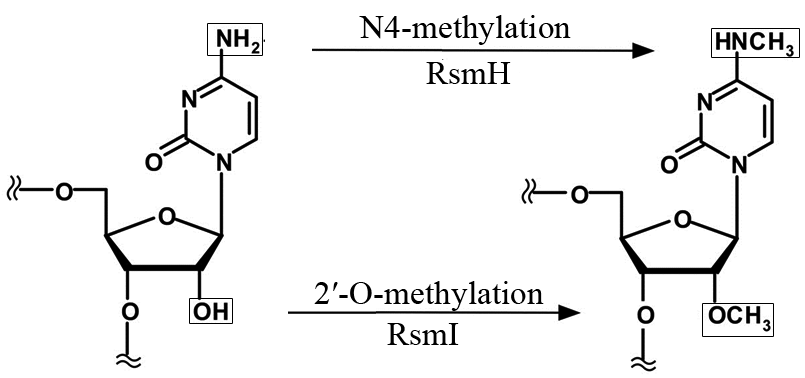

Supplement: S1 Fig — (TIF) [file pone.0163816.s001.tif]

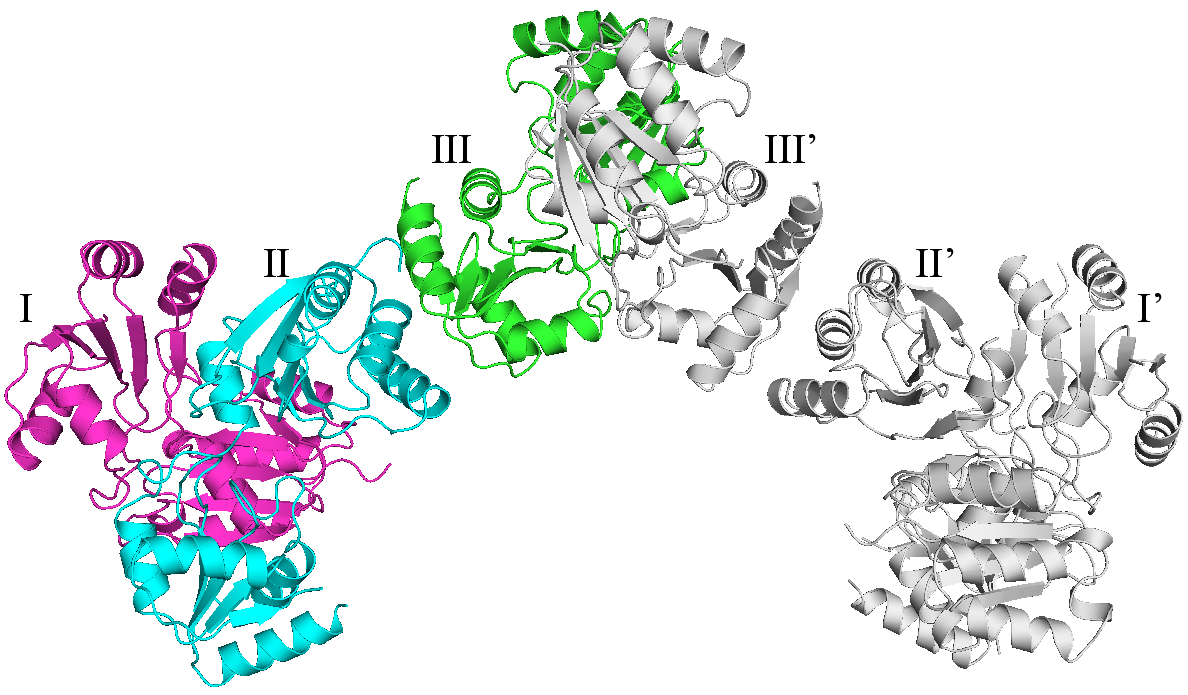

Supplement: S2 Fig — Three RsmI molecules (the subunits I’, II’ and III’) in another asymmetric unit are shown in gray. The subunits I and II (or I’ and II’) can form a compact homodimer. The subunit III forms another dimer with its crystallographic symmetry molecule III’. (TIF) [file pone.0163816.s002.tif]

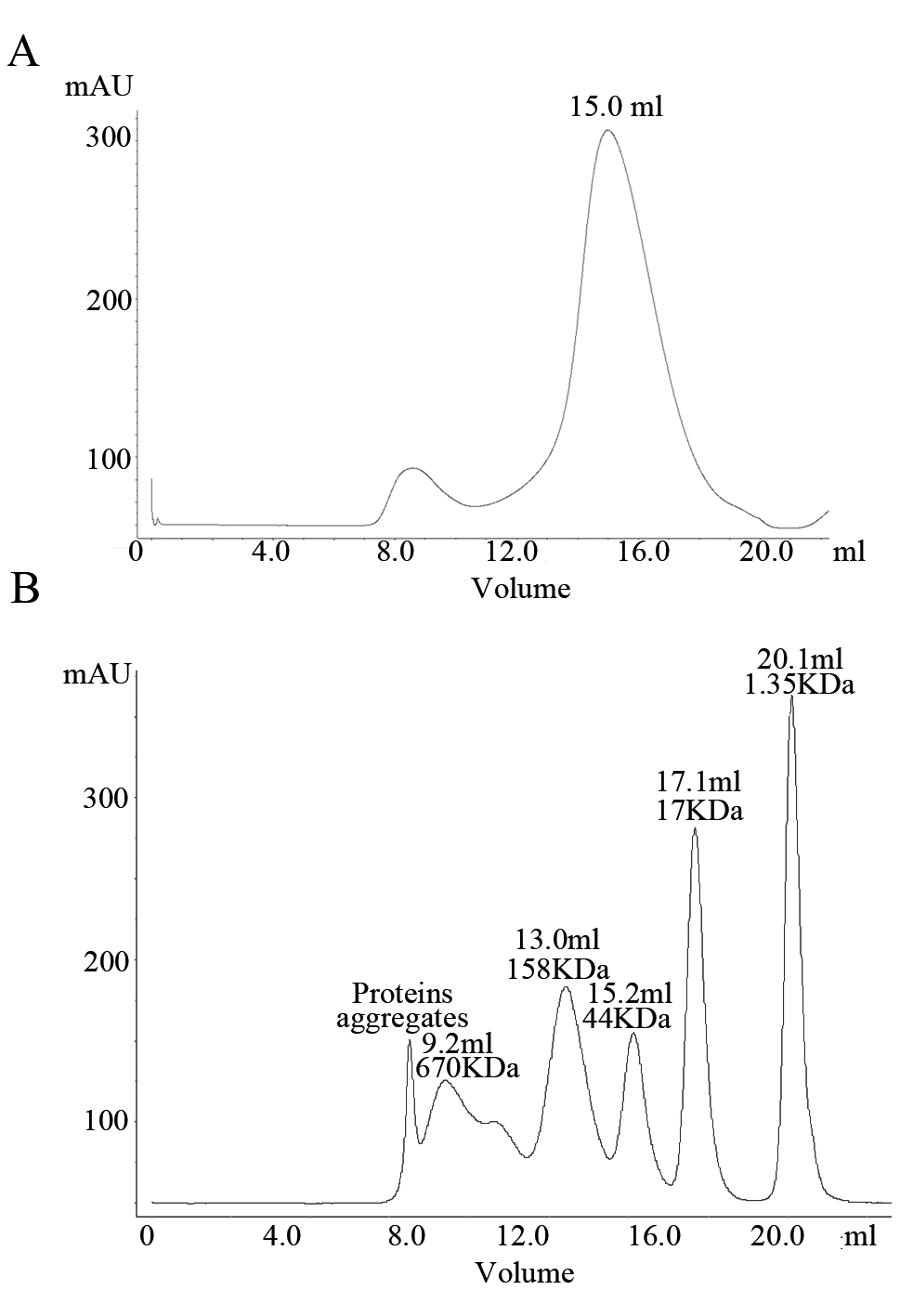

Supplement: S3 Fig — (TIF) [file pone.0163816.s003.tif]

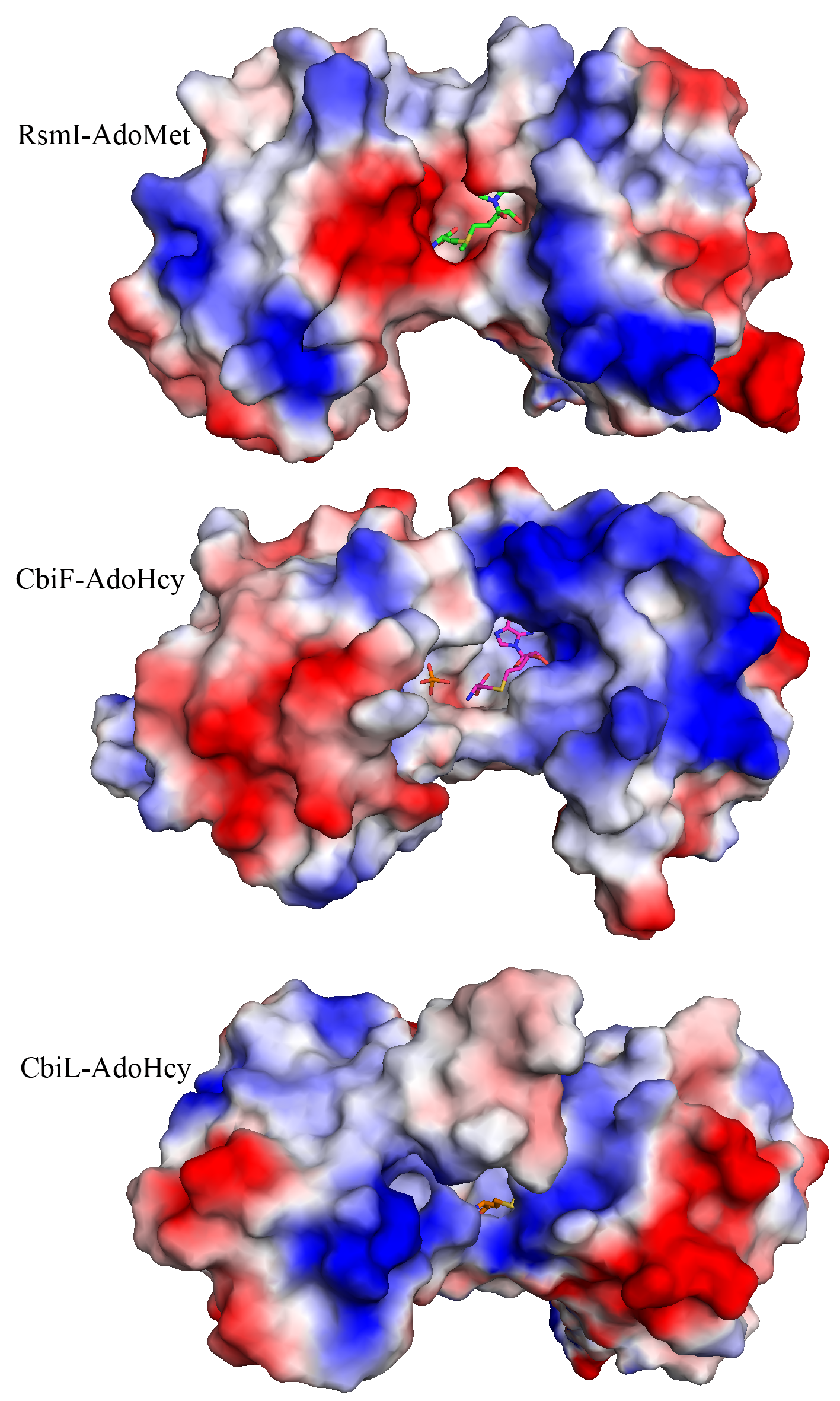

Supplement: S4 Fig — (TIF) [file pone.0163816.s004.tif]

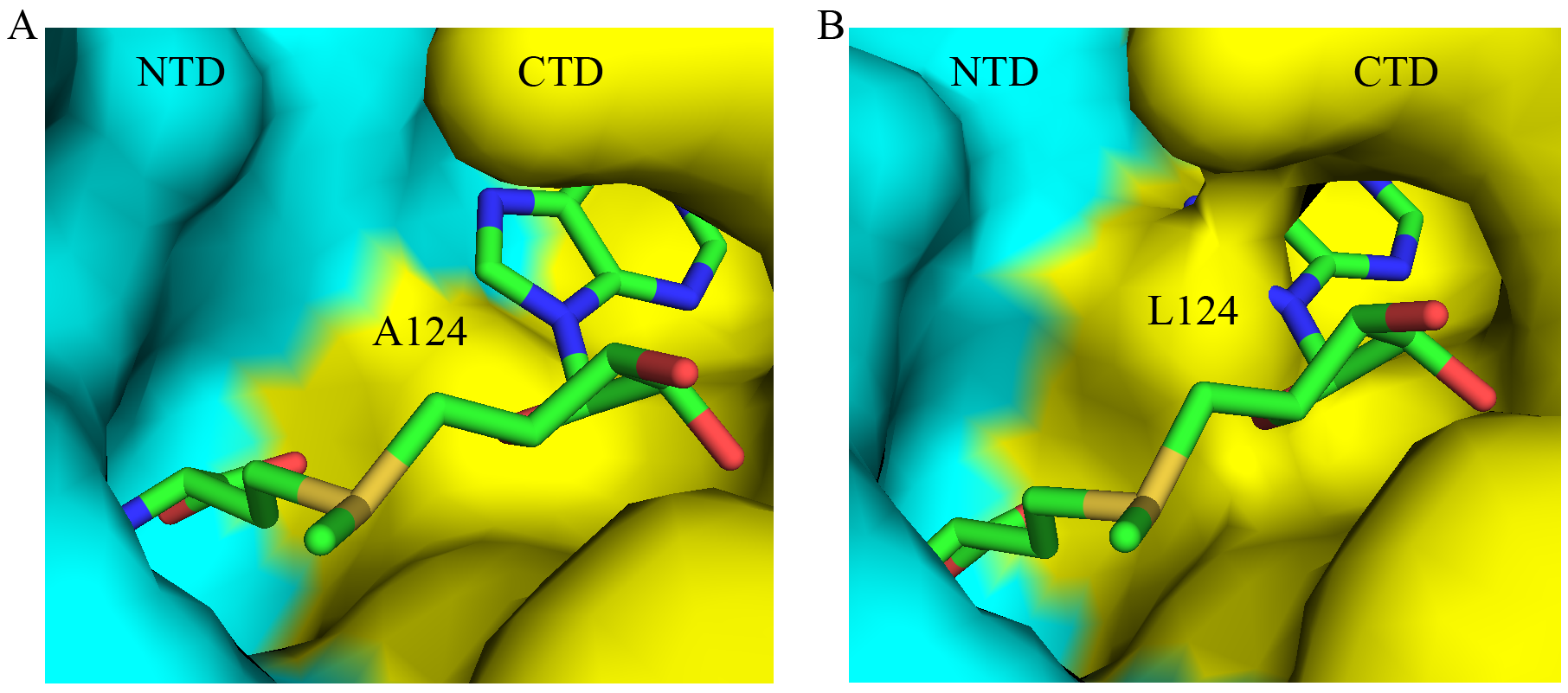

Supplement: S5 Fig — The adenosine ring of AdoMet shows obvious steric hindrance with L124 in the mutant model, causing its unfavorable binding to AdoMet. (TIF) [file pone.0163816.s005.tif]

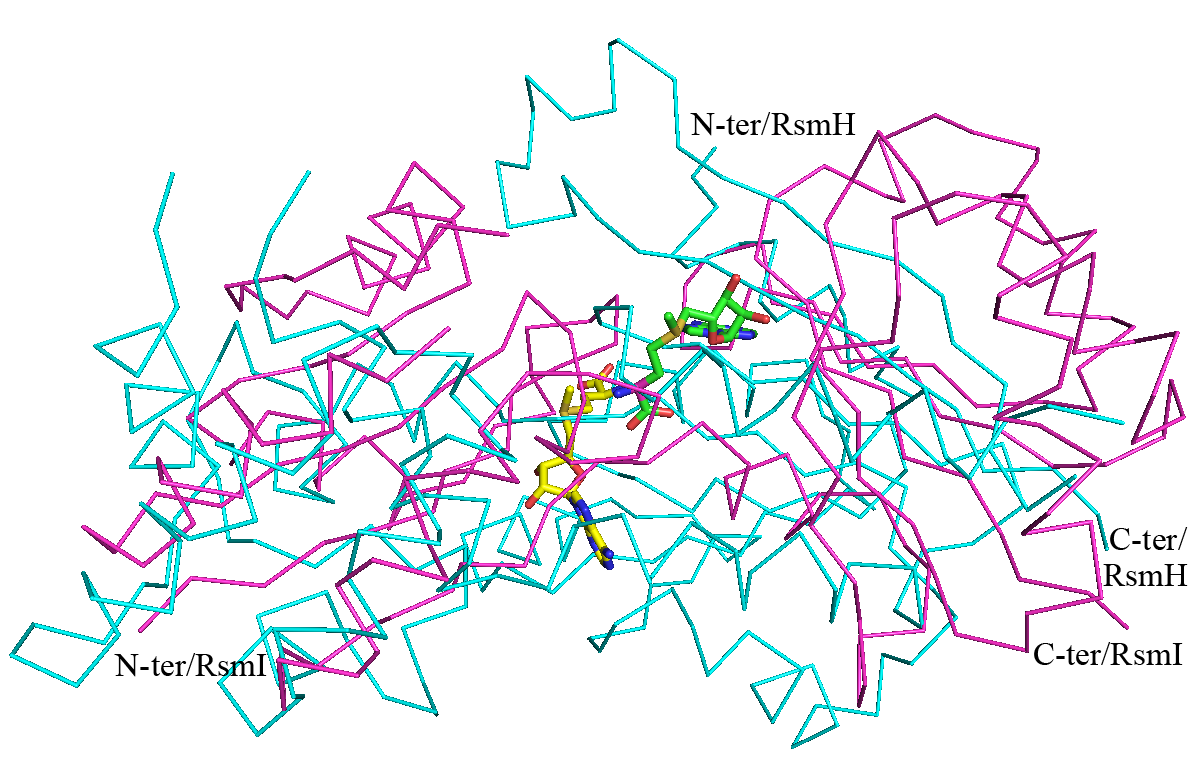

Supplement: S6 Fig — (TIF) [file pone.0163816.s006.tif]
