# Supplementary material for: Structural Insights into the Methylation of C1402 in 16S rRNA by Methyltransferase RsmI
Source: PLoS One. 2016 Oct 6;11(10):e0163816. doi: 10.1371/journal.pone.0163816 (PMC5053481; doi:10.1371/journal.pone.0163816)
Supplement: S1 Table — (DOCX) [file pone.0163816.s007.docx]

**Table S1.** The direct interactions (within 3.9 Å) between the two subunits of RsmI dimer analyzed by PDBe-PISA (<http://www.ebi.ac.uk/msd-srv/prot_int/pistart.html>).

| **Hydrogen bonds** | | | **Salt bridges** | | |
| --- | --- | --- | --- | --- | --- |
| **Subunit I** | **Distance**  **(Å)** | **Subunit II** | **Subunit I** | **Distance (Å)** | **Subunit II** |
| GLN  29[ N  ] | 3.32 | ALA  25[ O  ] | ARG  30[ NE ] | 2.92 | ASP  26[ OD1] |
| ARG  30[ N  ] | 2.96 | ALA  25[ O  ] | ARG  30[ NH2] | 3.43 | ASP  26[ OD1] |
| SER 136[ OG ] | 2.45 | LEU  97[ O  ] | ARG  30[ NE ] | 3.61 | ASP  26[ OD2] |
| TYR 191[ OH ] | 3.63 | GLU 111[ OE2] | ARG  30[ NH2] | 3.04 | ASP  26[ OD2] |
| ARG 110[ NH1] | 2.98 | ALA 131[ O  ] | ARG 164[ NH1] | 3.49 | GLU 142[ OE1] |
| TYR 141[ N  ] | 2.79 | PHE 139[ O  ] | ARG 164[ NE ] | 3.97 | GLU 142[ OE2] |
| PHE 139[ N  ] | 3.18 | TYR 141[ O  ] | ARG 164[ NH1] | 2.71 | GLU 142[ OE2] |
| ARG 115[ NH2] | 3.64 | GLU 203[ O  ] | ARG 164[ NH2] | 3.30 | GLU 162[ OE1] |
| ARG 110[ NH2] | 2.81 | ILE 205[ O  ] | ARG 115[ NH2] | 3.73 | GLU 203[ OE2] |
| ALA  25[ O  ] | 2.89 | ARG  30[ N  ] | ASP  26[ OD1] | 2.79 | ARG  30[ NE ] |
| ILE  27[ O  ] | 2.99 | GLN  29[ N  ] | ASP  26[ OD1] | 3.28 | ARG  30[ NH2] |
| LEU  97[ O  ] | 2.63 | SER 136[ OG ] | ASP  26[ OD2] | 3.68 | ARG  30[ NE ] |
| ALA 131[ O  ] | 3.05 | ARG 110[ NH1] | ASP  26[ OD2] | 3.02 | ARG  30[ NH2] |
| PHE 139[ O  ] | 2.77 | TYR 141[ N  ] | GLU 142[ OE1] | 3.89 | ARG 138[ NE ] |
| TYR 141[ O  ] | 3.12 | PHE 139[ N  ] | GLU 142[ OE1] | 3.83 | ARG 164[ NH1] |
| GLU 203[ O  ] | 3.47 | ARG 115[ NH2] | GLU 142[ OE2] | 2.76 | ARG 164[ NH1] |
| ILE 205[ O  ] | 2.54 | ARG 110[ NH2] | GLU 162[ OE1] | 3.52 | ARG 164[ NH2] |
|  |  |  | GLU 203[ OE2] | 3.55 | ARG 115[ NH2] |

| **Hydrogen bonds** | | | **Salt bridges** | | |
| --- | --- | --- | --- | --- | --- |
| **Subunit III** | **Distance**  **(Å)** | [**Subunit III’**](javascript:openWindow('pi_ipage_atom2.html',400,250);) | **Subunit III** | **Distance (Å)** | [**Subunit III’**](javascript:openWindow('pi_ipage_atom2.html',400,250);) |
| GLN  29[ N  ] | 3.26 | ALA  25[ O  ] | ARG  30[ NE ] | 2.90 | ASP  26[ OD1] |
| ARG  30[ N  ] | 2.85 | ALA  25[ O  ] | ARG  30[ NH1] | 3.55 | ASP  26[ OD1] |
| GLN  29[ N  ] | 3.58 | ILE  27[ O  ] | ARG  30[ NE ] | 3.46 | ASP  26[ OD2] |
| SER 136[ OG ] | 2.58 | LEU  97[ O  ] | ARG  30[ NH1] | 3.06 | ASP  26[ OD2] |
| ARG 110[ NH1] | 2.86 | ALA 131[ O  ] | ARG 164[ NH2] | 3.59 | GLU 142[ OE1] |
| TYR 141[ N  ] | 2.81 | PHE 139[ O  ] | ARG 138[ NE ] | 3.29 | GLU 142[ OE1] |
| PHE 139[ N  ] | 3.08 | TYR 141[ O  ] | ARG 138[ NH2] | 3.00 | GLU 142[ OE1] |
| ARG 115[ NH2] | 3.46 | GLU 203[ O  ] | ARG 164[ NE ] | 3.90 | GLU 142[ OE2] |
| ARG 110[ NH2] | 2.78 | ILE 205[ O  ] | ARG 164[ NH2] | 2.75 | GLU 142[ OE2] |
| ALA  25[ O  ] | 3.26 | GLN  29[ N  ] | ARG 138[ NE ] | 3.90 | GLU 142[ OE2] |
| ALA  25[ O  ] | 2.85 | ARG  30[ N  ] | ARG 138[ NH2] | 3.86 | GLU 142[ OE2] |
| ILE  27[ O  ] | 3.58 | GLN  29[ N  ] | ARG 164[ NH1] | 3.53 | GLU 162[ OE1] |
| LEU  97[ O  ] | 2.58 | SER 136[ OG ] | ARG 115[ NH2] | 3.62 | GLU 203[ OE2] |
| ALA 131[ O  ] | 2.86 | ARG 110[ NH1] | ASP  26[ OD1] | 2.90 | ARG  30[ NE ] |
| PHE 139[ O  ] | 2.81 | TYR 141[ N  ] | ASP  26[ OD1] | 3.55 | ARG  30[ NH1] |
| TYR 141[ O  ] | 3.08 | PHE 139[ N  ] | ASP  26[ OD2] | 3.46 | ARG  30[ NE ] |
| ILE 205[ O  ] | 2.78 | ARG 110[ NH2] | ASP  26[ OD2] | 3.06 | ARG  30[ NH1] |
|  |  |  | GLU 142[ OE1] | 3.29 | ARG 138[ NE ] |
|  |  |  | GLU 142[ OE1] | 3.00 | ARG 138[ NH2] |
|  |  |  | GLU 142[ OE1] | 3.59 | ARG 164[ NH2] |
|  |  |  | GLU 142[ OE2] | 3.90 | ARG 138[ NE ] |
|  |  |  | GLU 142[ OE2] | 3.86 | ARG 138[ NH2] |
|  |  |  | GLU 142[ OE2] | 3.90 | ARG 164[ NE ] |
|  |  |  | GLU 142[ OE2] | 2.75 | ARG 164[ NH2] |
|  |  |  | GLU 162[ OE1] | 3.53 | ARG 164[ NH1] |
|  |  |  | GLU 203[ OE2] | 3.62 | ARG 115[ NH2] |
